# UPLC-ESI/MS-MS-based phytochemical analysis of *Schinopsis balansae* leaf extract and anti-skin ageing properties supported with *in silico* molecular docking experiments

**DOI:** 10.1038/s41598-026-50709-6

**Published:** 2026-05-15

**Authors:** Heba A. S. El-Nashar, Ayman M. Al-Qaaneh, Mahmood A. Al-Azzawi, Abdalrahman Tarek, Esraa A. Elhawary, Naglaa S. Ashmawy

**Affiliations:** 1 Department of Pharmacognosy, Faculty of Pharmacy, Ain Shams University, Abbassia, Cairo, 11566 Egypt; 2 Department of Pharmacognosy, Faculty of Pharmacy, Modern University for Technology & Information, Cairo, 11571, Egypt; 3 Faculty of Allied Medical Sciences, Al-Balqa Applied University (BAU), Al- Salt, 19117 Jordan; 4 Department of Forensic Science, College of Science, Al-Karkh University of Science, P.O. Box 10081, Baghdad, Iraq; 5 Department of Pharmaceutical Chemistry, Faculty of Pharmacy, Ain Shams University, Abbassia, Cairo, 11566 Egypt; 6 Department of Pharmaceutical Sciences, College of Pharmacy, Dubai Medical University, P.O. Box 4184, Ajman, United Arab Emirates

**Keywords:** Anacardiaceae, Flavonoids, HPLC-ESI/MS-MS, *Schinopsis balansae*, Tannins, Collagenase, Elastase, Biochemistry, Chemical biology, Chemistry, Computational biology and bioinformatics, Drug discovery, Plant sciences

## Abstract

*Schinopsis balansae* is a tannin-rich tree belonging to the family Anacardiaceae, native to the Gran Chaco region of Argentina, and cultivated in Egypt. This study was performed to characterize the bioactive compounds of the 80% methanol extract of *S. balansae* leaves using HPLC-ESI/MS-MS technique, coupled with the assessment of *in vitro *anti-aging enzyme inhibition assays. Further, total phenolic and flavonoid content and molecular docking studies were conducted. High performance liquid chromatography-electrospray ionization coupled with mass fragmentation (HPLC-ESI/MS-MS) was used to tentatively identify the phyto-constituents of *S. balansae* leaf extract. The phenolic and flavonoid contents were assessed as gallic acid equivalents (GAE) and rutin equivalents (RE). Further, the anti-skin aging potential was investigated *via* assessment of elastase and collagenase inhibition assays. The in silico molecular docking studies were conducted on 7EST elastase and 2TCl collagenase. The HPLC/MS analysis revealed the identification of twenty-two compounds of different chemical classes like flavonoids, phenolic acids, and tannins. The total phenolic and flavonoid contents were 399.29 ± 13.23 µg gallic acid equivalent/mg and 8.25 ± 0.63 µg rutin equivalent/mg, respectively. The extract exhibited significant collagenase (IC_50_ = 48.08 ± 3.42 µg/mL) and elastase (IC_50_= 18.02 ± 1.57 µg/mL) inhibitory activities, suggesting its potential in combating skin aging. *In silico* molecular docking studies validated the interactions of major compounds like quercetin-*O*-benzoyl-hexoside and 5-hydroxy-7,4'-dimethoxy-6,8-dimethyl homo-isoflavone with these enzymes. This study provides a scientific rationale for the anti-skin-aging potential of *S. Balansae* leaf extract, supporting its utilization in innovative anti-wrinkle formulations.

**Supplementary Information:**

The online version contains supplementary material available at 10.1038/s41598-026-50709-6.

## Introduction

Skin wrinkle is a complex process associated with age-dependent decline in the skin cell functions, resulting in visible signs on the surface of the skin^1^. There are a variety of clinical signs associated with skin aging, including irregular dryness, dark/light pigmentation, sallowness, deep furrows or severe atrophy, telangiectasis, premalignant lesions, laxity, leathery appearance, and so on^2^. Several scientific studies have indicated that skin wrinkles are associated with a degradation of the extracellular matrix (ECM), which is associated with increased dermal enzymatic activities including collagenase, elastase, hyaluronidase and matrix metaloprotease-1 (MMP-1)^3^. A number of natural skin care ingredients have the potential of inhibiting these enzymes in aged skin and would also play a role in restoration of the normal skin function and prevention or/treatment of wrinkles^4^.

*Schinopsis balansae* commonly known as ‘‘quebracho colorado’’ is a native tree from South America and is very valued for its high content of tannins^5,6^. Phytochemistry of this tree has been investigated only regarding its heartwood rich in tannins^7^. These tannins have a wide range of applications in various fields^8,9^. They have been used for microcapsules and nanoemulsions generation^10^, for Nickel/Carbon nanocomposites production^11^ and for the synthesis of antifouling^12–14^ & anticorrosive coatings^15^. Furthermore, these tannins were applied in the synthesis of nanoparticle with antimicrobial potential^16^ and nanofunctionalization of protective antifungal coatings^5,17,18^. Moreover, these tannins were utilized as a coagulant and flocculant agent^19^, as building blocks for thermoset resins^20^. These tannins showed an antiparasitic^21^, antimutagenic and antioxidant activities^22^. In addition to its wide application in leather tannery^20^ and for water treatment^23–26^.

Regarding the leaves of *S. balansae*, few studies reported its phytoconstituents, Ficoseco et al. reported the leaf constituents, mainly composed of a mixture of four 3-*n*-heptadec(en)ylcatechols (PALK), with significant antifungal activity^7^. In another study, García et al. explored the condensed tannins isolated from the leaves of *S. balansae* with investigation of its in vitro true digestibility and nutritional value^27^. While the phytochemical profile of *S. balansae* leaves is not extensively studied, the available literature reports that most studies have focused on its tannin-rich heartwood. Safety evaluations specifically for the leaf extract are scarce. However, *Schinopsis* species’ tannins have been used in water treatment and leather industries, implying a generally safe profile when appropriately processed^28^. 

Phenolics of *Schinopesis* plants have been categorized as condensed tannins^22^. Flavonoids are the predominant imperative group of phenolics and signify two thirds of dietary phenolics^29^. In recent years, awareness and interest have increased regarding the potential health benefits of phenolic compounds due to significant antioxidant potential^30^. Phenolics as natural antioxidants could inhibit diseases associated with oxidative damage such as skin aging, Alzheimer, diabetes mellitus, coronary heart diseases and cancer^31^ Particularly, green tea polyphenols are commonly used as effective anti-skin aging agents in skin care preparations due to their inhibitory properties against collagenase and elastase activity, apparently through non-covalent binding^32^.

Our study aimed to investigate the phytoconstituents of 80% methanol extract of *S. balansae* leaves using UPLC-ESI/MS-MS analysis. Further, we evaluated the inhibitory activity of the leaf extract against skin aging enzymes including collagenase and elastase *via in vitro* assays and *in silico* molecular docking studies.

## Materials and methods

### Plant material

The fresh leaves of *S. balansae* were collected in in May 2022 from El-Zohrya Garden beside Cairo Tower, Cairo, Egypt, N 30°02’49.218” E 31°13’32.898”. The plant parts were kindly identified and authenticated by Mrs. Therese Labib, Botanical Consultant at El-Orman Garden, Giza, Egypt. Voucher specimens of the plant parts with code of PHG-P-SB-413 was kept at the Herbarium of Pharmacognosy Department, Ain Shams University, Abbassia, Cairo, Egypt.

The fresh leaves (250 g) were air-dried at room temperature and ground into coarse powder. The powdered leaves were exhaustively extracted with 80% methanol (v/v) in distilled water using maceration. Specifically, the plant material was soaked in 3 L of 80% methanol for 72 h at room temperature with occasional stirring. This process was repeated three times to ensure maximum extraction of phytoconstituents. The combined extracts were filtered through Whatman No.1 filter paper and concentrated under reduced pressure at 45 °C using a rotary evaporator until complete dryness.

### Determination of total phenolic and flavonoid contents

The total phenolic and flavonoid contents (TPC and TFC) were measured according to the previous described procedures^33,34^. The results of this study were expressed as gallic acid equivalents (GAE) and rutin equivalents (RE) for TPC and TFC, respectively.

### UPLC-ESI/MS-MS analysis conditions

The metabolic profiling of *S. balansae* extract was evaluated using high-performance liquid chromatographic (HPLC) analysis joined with an ESI-MS/MS spectrometer detector^35^. The extract (100 *µ*g/mL) was dissolved in methanol (HPLC-grade), then filtered *via* a membrane disc (0.20 *µ*m). Then, the filtrate (10 *µ*L) was injected into an HPLC-ESI-MS/MS system. The used HPLC instrument has the following specifications: Waters^®^ equipped with a reversed-phase C-18 column (ACQUITY UPLC-BEH C-18, particle size ~ 1.7 μm, dimensions = 2.1 × 50 mm). Prior to injection, the mobile phase was filtered through a membrane disc filter (0.2 *µ*m) and sonicated. The elution run took 35 min using gradient elution (water (A) and methanol (B), both acidified with 0.1% formic acid) with a flow rate of 0.2 mL/min. The complete chromatographic gradient program was applied as follows: (0–3 min, 10% B; 3–25 min, 10–100% B; 25–30 min, 100% B; 30–31 min, 100–10% B; 31–35 min, 10% B). Mass spectrometry analysis was performed on a Waters^®^ XEVO TQD triple quadrupole instrument (Waters^®^ Corporation, Milford, MA, USA). The vacuum pump was provided by Edwards^®^, USA. Positive and negative ions were acquired using an electrospray ionization (ESI) source with the following ionization source parameters: capillary voltage, 3.0 kV in both positive and negative modes; cone voltage, 30 V; source temperature, 150 °C; desolvation temperature, 440 °C; nebulization and desolvation gas (nitrogen) flow, 900 L/h; and cone gas flow, 50 L/h. The mass spectra were obtained using MassLynx 4.1 software at an ESI range *m/z* of 100–1000. For the MS/MS analysis, fragmentation of the precursor ions was carried out using Data Dependent Acquisition (DDA) mode. Nitrogen was utilized as the collision gas in the triple quadrupole analyzer, with applied collision energies ranging from 15 to 45 eV. In order to tentatively identify the obtained mass spectra, the peak retention time (R_t_) and their fragmentation outline were compared with the reported data in the literature.

### Determination of collagenase inhibitory activity

Collagenase inhibition was qualified spectrophotometrically by means of Abcam’s collagenase activity assay Kit (#ab196999) based on a previously designated technique with minor changes^36^. Collagenase (EC 3.4.24.3, Sigma, USA) was sourced from *Clostridium histolyticum* according to the provided protocol and dissolved in 50 mM Tricine buffer (pH = 7.5, 10mM CaCl_2_+400mM NaCl). The synthetic substrate was N-[3-(2-furyl) acryloyl]-Leu–Gly–Pro–Ala (FALGPA) in 2 mM Tricine buffer. In 96-well plates, serial concentrations of the plant extract (0.1–100 µg/mL) were incubated at 25 °C with the ready collagenase in Tricine buffer for 15 min. After that, the synthetic substrate (40 µL) was added to the different extract concentrations. The absorbance values were measured at 345 nm for 10–15 min away from light at 37 °C *via* a microplate reader (TECAN, Inc., Durham, NC, USA). The standard was piroxicam, whilst the negative control included water. The collagenase inhibition (%) was calculated according to the following formula: %inhibition= [1−( sample absorbance / control absorbance) ×100]. Then, the concentration inhibiting 50% of the enzyme (IC_50_), was constructed from the graph plots of the concentration-response curve for each plant concentration using GraphPad Prism software version 7 (San Diego, CA, USA).

### Determination of elastase inhibitory activity

The elastase inhibition activity was examined spectrophotometrically using EnzChek^®^ Elastase Assay Kit (#E-12056) as stated by previously defined technique with minor changes^37^. For preparation of stock solution from elastase (EC3.4.21.36), it was obtained from pancreatic porcine, was dissolved in sterile water. N-succinyl-Ala–Ala–Ala–p-nitroanilide (AAAPVN, 0.8 mM) was used as a synthetic substrate and dissolved in Tris-HCL buffer (pH = 8, concentration = 1.6mM). In 96-well microtitre plate, serial concentrations of plant extract (0.1 to 100 µg/mL) was incubated at 25 °C with the prepared elastase in Tris-HCL buffer for 15 min. Afterward, the synthetic substrate was added to the prepared concentrations to start the reaction to obtain a final mixture with a total volume 250 µL. The absorbance values were measured at 450 nm for 5–15 min with light protection at 37 °C *via* a microplate reader (TECAN, Inc., Durham, NC, USA). Epigallocatechin gallate (EGCG) was used as a positive control, whilst the negative control was water. The inhibitory activity of elastase (%) was calculated according to the following formula: % inhibition= [1−(Sample absorbance/Control absorbance) ×100]. After that, we identified the concentration inhibiting 50% of the enzyme (IC_50_), from the graph plots of the concentration-response curve for each extract concentration using GraphPad Prism software version 7 (San Diego, CA, USA).

### *In silico* molecular docking studies

Molecular docking analysis of the major identified compounds of the extract was accomplished using Discovery Studio 2.5.5 and the results were displayed *via* Discovery Studio Visualizer 2016. The co-crystallized 3D structures of the target proteins collagenase (PDB ID: 2TCL) and elastase (PDB ID: 7EST) were downloaded from PDB (www.rcsb.org). The prepare protein protocol was used to prepare the downloaded target proteins. This includes protonation of the downloaded proteins, addition of the missing loops, insertion of missing atoms in the amino acids of the protein and standardizing their names, and application of forcefield CHARMm. Tested compounds were docked in the same active pocket of the co-crystallized ligands. Prepare ligand protocol was used to prepare the investigated compounds. Parameters were adjusted to remove any duplicates, fix bad valencies, generate tautomers, add hydrogens, and minimize energy. The type of docking algorithm that was chosen is CDOCKER. The input site sphere coordinates for 2TCL (collagenase) are (74.1185, 8.69847, 8.94462) with a radius of 9.18119 Å. Regarding 7EST (elastase), the coordinates are (14.5994, 47.1026, -0.0567262) and the radius of the sphere is 9.84239 Å. The tested compounds including 5-hydroxy-7,4’-dimethoxy-6, 8-dimethyl homo-isoflavone (PubChem CID 5386259; CAS 34086-51-6), scopoletin (PubChem CID 5280460; CAS 92-61-5), jatrorrhizine (PubChem CID 72323; CAS 3621-38-3), oleanolic acid (PubChem CID 10494; CAS 508-02-1), eburicoic acid (PubChem CID 73402; CAS 560-66-7), EGCG (PubChem CID 65064; CAS 989-51-5), and piroxicam (PubChem CID 54676228; CAS 36322-90-4) were arranged according to their -CDOCKER score. The higher -CDOCKER score the better. Docking was validated by redocking of the co-crystallized ligands and calculating RMSD. RMSD for 7EST was 1.1778 and for 2TCL, it was 1.9239. RMSD in both cases was less than 2.

### Statistical analysis

The enzyme inhibition assays were performed in triplicates, and the obtained values are indicated as mean ± SD. For collagenase, and elastase inhibitory aspects, the IC_50_ was attained from the graph plots of the concentration-response curves at each extract concentration *via* Graph Pad Prism Software Version 7 (San Diego, CA, USA). The IC_50_ is the concentration of the plant extract required to inhibit 50% of the enzyme activity under the functional assay conditions.

## Results and discussion

### Estimation of total phenolic and flavonoids contents of *S. balansae* leaf extract

The plant-derived phenolic compounds represent an essential class of phytochemicals due to their richness with hydroxyl groups that provide scavenging capacity^38^. The Phenolic compounds are divided into several types; foremost among these are the flavonoids which exhibit potent antioxidant properties^39^. Flavonoids are naturally occurring compounds with positive impact on human well-being^31^. Different studies reported a wide range of antioxidant, vasodilator, anti-inflammatory, anticancer, and anti-allergic activities of flavonoids^40^. Specifically, flavonoids have been revealed to be highly active hunters for reactive oxygen species (ROS), containing singlet oxygen, and various reactive oxidizing agents associated with several diseases like aging manifestations, cancer, diabetes, cataract and so on^41^. Interestingly, our findings showed that the total phenolic content was about 399.29 ± 13.23 µg GA/mg extract. Also, the total flavonoid content was 8.25 ± 0.63 µg rutin eq/mg extract. With the same line of our results, *Schinopesis* plants’ extracts are used in tannery due to their high concentration of phenolics^20^. A certain study in Mexico found that the total phenolic content of wastewaters of *S. balansae* contains about 621 mg catechin equivalent/g extract, being gallic and protocatechuic acids the predominant components^22^. A study reported isolation of sixteen antioxidant phenolic compounds containing galloyl derivatives, from *S. brasiliensis* stem barks^42^. Further, high content of phenolic compounds (tannins = 455.81 ± 50.41 mg TAE/g; flavonoids = 11.29 ± 0.94 mg RE/g) were found in the methanol extract of *S. brasiliensis* leaves^43^.

### UPLC-ESI/MS-MS metabolic analysis of *S. balansae* leaf extract

**Fig. 1:**
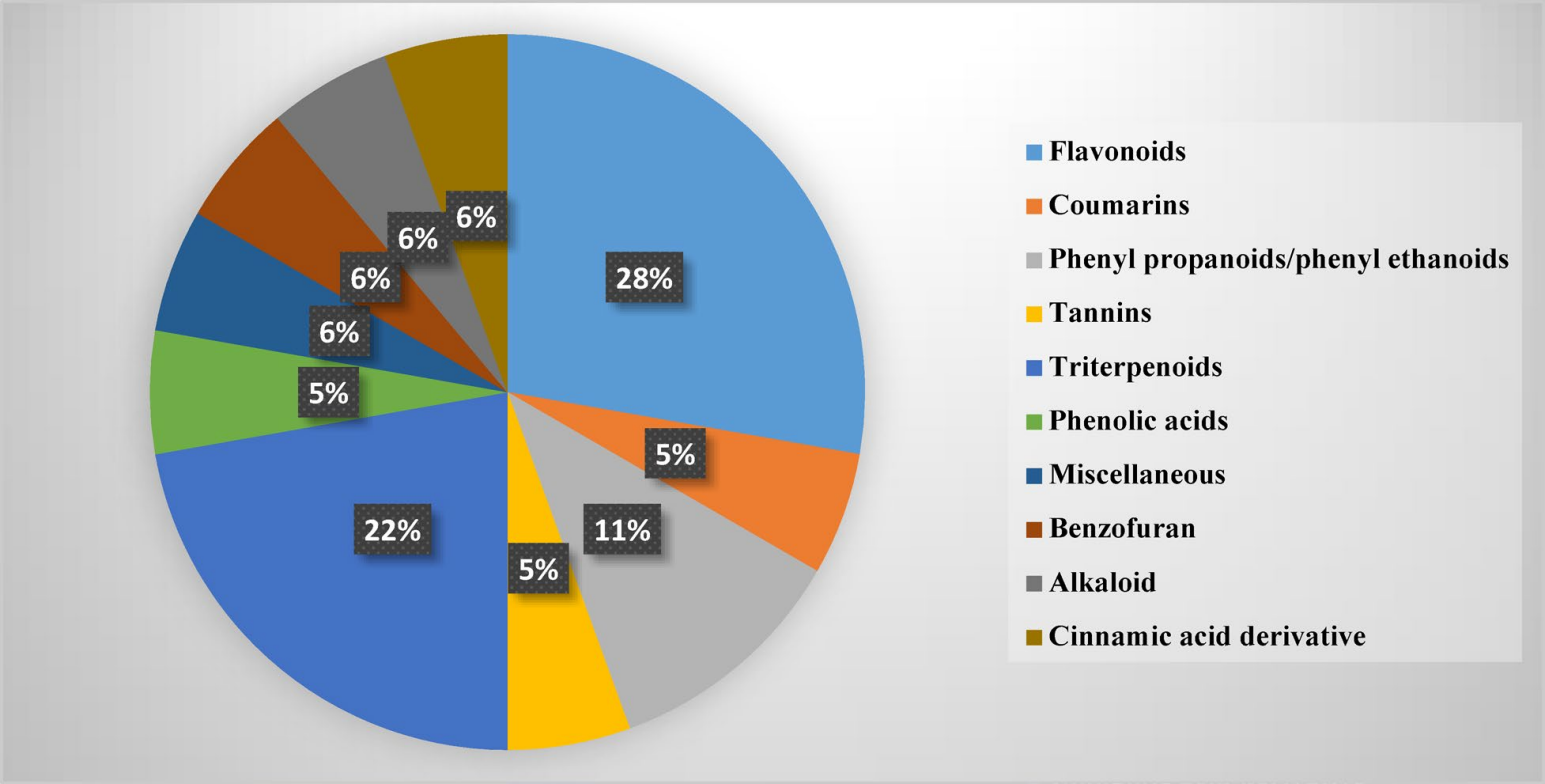
Bar chart representing the classes of the tentatively identified secondary metabolites from *S. balansae.*

As illustrated in Figure S1 and Table 1, twenty-two secondary metabolites were tentatively in the 80% methanol extract of *S. balansae* leaves. The identified components were abundantly from different classes viz. flavonoids, phenolic acids, phenyl propanoids/phenyl ethanoids, di- and triterpenoids, alkaloids, tannins, coumarins and others (Fig. 1). The identification of these components was achieved through comparison of their molecular ion peaks and fragmentation patterns to the reported compounds in relevant literature. The tentatively identified secondary metabolites can be discussed as follows.

Compounds 3 presented a deprotonated peak at *m/z* 343(345) with MS/MS at *m/z* 299, 281, 213, 191 and 169 and molecular formula C_14_H_16_O_10_ thus it was tentatively defined as galloyl quinic acid (3.01%) as shown in Figure S2^44,45^. This compound exhibited a precursor ion at *m/z* 687 [2 M-H]^−^, which dissociated to yield a highly abundant monomeric ion at *m/z* 343. Further fragmentation of the *m/z* 343 ion produced major diagnostic product ions at *m/z* 191 [quinic acid-H]^−^ and *m/z* 169 [gallic acid - H]^−^. The transition from *m/z* 343 to 191 corresponds to a characteristic neutral loss of 152 Da, which is definitively indicative of the cleavage of a galloyl moiety. Additionally, an ion at *m/z* 125 was observed, representing the neutral loss of CO_2_ (44 Da) from the gallic acid fragment.

A flavone glycoside was traced at *m/z* 565 (positive mode only, C_39_H_32_O_15_, 14.06%) with fragments detected at *m/z* 301, 255, 235 and 191 which was identified as justicialoside A (Figure S3) (identified before from family Anacardiaceae)^46^. Moreover, compound 22 had a parent peak at *m/z* 339 and daughters at *m/z* 325, 311, 265, 235, 186 and 145 which was then defined as 5-hydroxy-7,4’-dimethoxy-6, 8-dimethyl homo-isoflavone (4.19%)^47^. In addition to that, quercetin was a common aglycone appearing at *m/z* 301 for compounds 5, 6 and 16. The aforementioned components presented their peaks at *m/z* 585, 599 and 568 (positive mode), respectively which lead to their tentative identification as quercetin 7-*O*-galloyl-hexoside^48^, quercitrin-*O*-gallate^49^ and quercetin-*O*-benzoyl-hexoside^50^, respectively.

In addition to that, one coumarin (Table 1) was detected at *m/z* 191(193) with fragments at *m/z* 185, 159 and 140 which was tentatively assigned to scopoletin (identified before from family Anacardiaceae)^51^. Moreover, one major (area%= 68.41%) phenyl propanoid peak was detected at *m/z* 421(423) and its fragments at *m/z* 365, 343, 293, 235, 191, 183 and 175 where the peak at *m/z* 191 was due to loss of the quinic acid moiety thus it was tentatively identified as malonyl-coumaroyl-quinic acid (Figure S4)^52^. Similarly, one phenyl ethanoid was detected at *m/z* 639 (positive mode) with MS/MS at *m/z* 595, 486, 428, 387, 345, 332, 291, 277 and 241 and it was defined as leucosceptoside A^53^.

A tannin peak was defined as compound 8 with its peak at [M-H]^−^ *m/z* 561 and fragments at *m/z* 502, 473, 409, 403, 397, 391, 354, 326, 229 and 207 where the two fragments at m/z 409 and 391 were reported before for the cleavage of the cinnamoyl moiety followed by loss of water molecule thus this compound was assigned to quebracho dimer (previously identified from genus *Schinopsis*)^54^. Moreover, one diterpenoid and three triterpenoids were defined (Table 1).

**Fig. 2:**
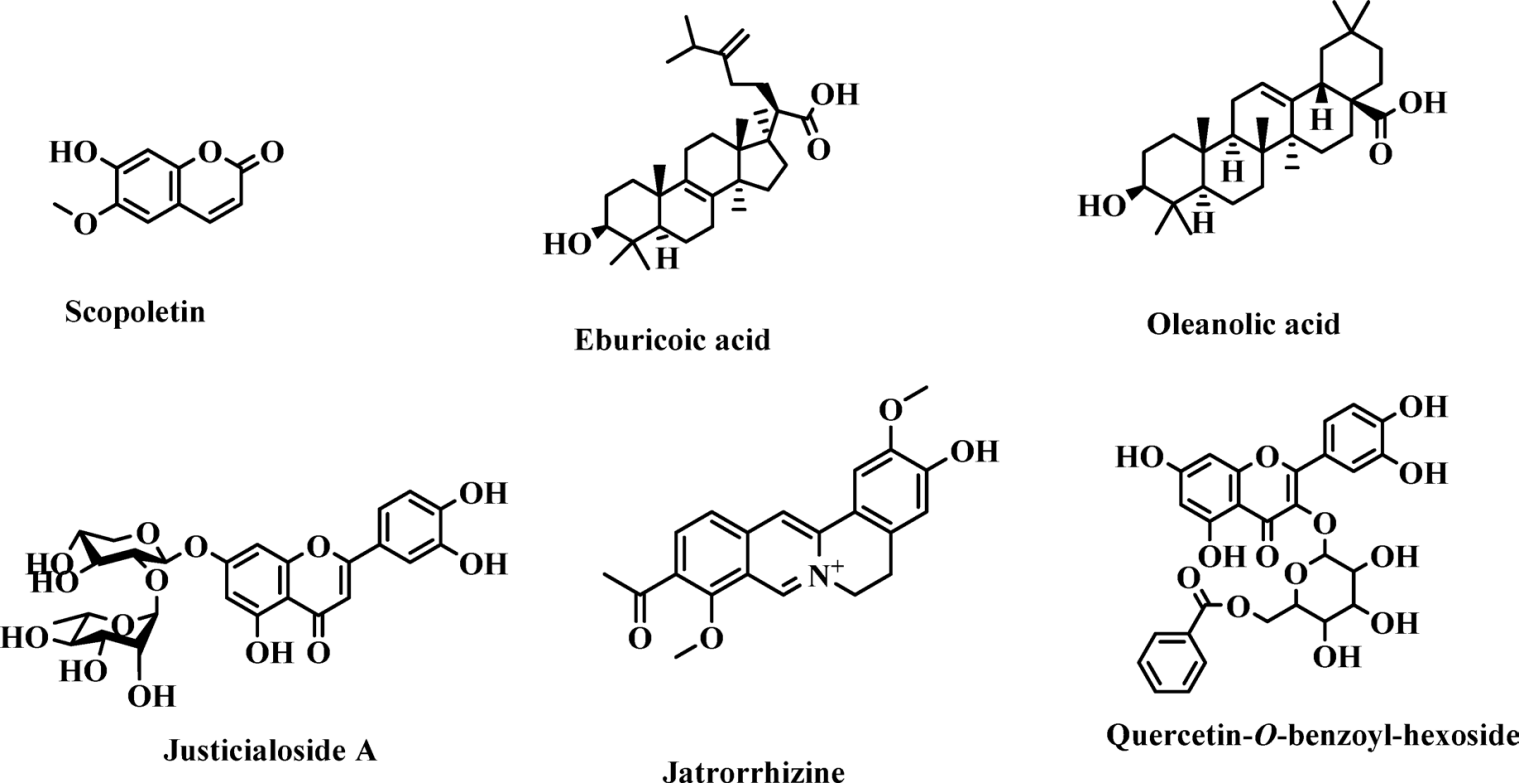
Chemical structures of major metabolites identified in *S. balansae* leaf extract *via* UPLC-ESI/MS-MS.

Compound 8 was defined as an oleanolic acid derivative (*m/z* 519)^55^ while compound 21 was tentatively assigned to oleanolic acid which presented a deprotonated peak at *m/z* 455(457) and fragments at *m/z* 425, 391, 339, 317, 292, 248, 207 and 190 where the fragments at *m/z* 248 and 207 are due to the cleavage of ring C as displayed in Figure S6^56^. Moreover, compound 13 presented a parent peak at *m/z* 490 [M + H+H_2_O] in positive mode with fragments at *m/z* 420, 398, 335, 279, 217 and 191 and it was defined as eburicoic acid (Figure S6) (previously reported from family Anacardiaceae)^57^. In addition to that, several other secondary metabolites were traced and identified and had deprotonated peaks at *m/z* 325, 333, 607, 557 and 413, respectively thus they were tentatively defined as *p*-coumaroyl-*O*-hexoside (phenolic acid)^44^, salvianolic acid K (benzofuran)^58^ and isopentyl dihexose^59^, respectively (Table 1). *Schinopesis* plants’ residues were demonstrated to be an outstanding source of phenolic acids and for research and industrial uses^22^. All chemical structures of the identified compounds are displayed in Fig. 2.

**Table 1:** UPLC-ESI/MS-MS based characterization of phytoconstituents of 80% methanol extract *S. balansae* leaves in negative and positive ionization modes.

No.	Compound name	Molecular formula	Class	*R*_t_ (min.)	Area %	[M-H]^−^ (m/z)	[M + H]^+^ (m/z)	MS/MS fragments	Reference(s)
1	*Unidentified*	–	-----	0.22	1.02	–	187	-----	-----
2	Scopoletin^f^	C_10_H_8_O_4_	Coumarin	0.76	**8.13** **(8.99)**	191	193	185, 159, 140	^51^
3	Galloyl quinic acid	C_14_H_16_O_10_	Cinnamic acid deriv.	1.01	**3.01** (0.44)	343	345	299, 281, 213, 191, 169	^44, 45^
4	Malonyl-coumaroyl-quinic acid	-----	Phenyl propanoid	2.20	**68.41**	421	423	365, 343, 293, 235, 191, 183, 175	^52^
5	Quercetin 7-*O*-galloyl-hexoside	C_28_H_24_O_16_	Flavonoid	6.42	1.53	585	–	301, 153, 132	^48^
6	Quercitrin-*O*-gallate	C_28_H_24_O_15_	Flavonoid	7.15	1.18	599	–	569, 465, 413, 405, 368, 335, 301, 285, 187	^49^
7	Quebracho dimer^*^	-----	Tannin	13.92	0.89	561	–	502, 473, 409, 403, 397, 391, 354, 326, 229, 207	^54^
8	Oleanolic acid derivative	-----	Triterpenoid	14.16	0.49	–	519	487, 395, 357, 324, 279, 222	^55^
9	*Unidentified*	-----	-----	15.08	1.48	–	520	473, 415, 317, 280, 219, 204, 168	-----
10	*p*-Coumaroyl-*O*-hexoside	C_15_H_18_O_8_	Phenolic acid	16.47	1.99	325	-	119	^44^
11	Leucosceptoside A	C_30_H_38_O_15_	Phenyl ethanoid glycoside	17.47	0.52	–	639	595, 486, 428, 387, 345, 332, 291, 277, 241	^53^
12	*Unidentified*	-----	-----	18.27	2.91	–	560	396, 302, 265, 222	-----
13	Eburicoic acid^f^	C_31_H_50_O_3_	Triterpenoid	19.43	**8.63**	–	490 [471 + H_2_O]	420, 398, 335, 279, 217, 191	^57^
14	Justicialoside A^f^	C_26_H_28_O_14_	Flavone glycoside	20.01	**14.06**	–	565	301, 255, 235, 191	^46^
15	*Unidentified*	-----	-----	20.96	0.79	–	384	356, 311, 307, 279, 251, 233, 182	-----
16	Quercetin-*O*-benzoyl-hexoside	C_28_H_23_O_13_	Flavonoid	22.09	**5.25**	–	568	439, 406, 323, 311, 301, 250, 214	^50^
17	Oleanolic acid	C_30_H_48_O_3_	Triterpenoid	22.29	**10.67** **(8.22)**	455	457	425, 391, 339, 317, 292, 248, 207, 190	^56^
18	dehydrated Oleanolic acid	C_30_H_48_O_3_	Triterpenoid	25.01	**5.17**	–	439 [M + H-H_2_O]^+^	333, 311, 305, 293	^56^
19	Salvianolic acid K	C_27_H_24_O_13_	Benzofuran	25.20	0.74	–	557	-----	^58^
20	Isopentyl dihexose	-----	Miscellaneous	25.63	2.18	–	413	393, 294, 214, 194	^59^
21	Jatrorrhizine	C_20_H_20_NO_4_+	Alkaloid	31.05	**3.67**	–	339	-----	^60^
22	5-Hydroxy-7,4'-dimethoxy-6, 8-dimethyl homo-isoflavone	-----	Flavonoid	31.09	**4.19**	339	-	325, 311, 265, 235, 186, 145	^47^
	% Identification			100% (negative mode) 64.56% (positive mode)					

^*^for compounds isolated before from genus *Schinopsis*, ^f^ for compounds isolated before from family Anacardiaceae. Bold numbers are for the major components and area % between brackets are for the positive mode.

### Assessment of collagenase and elastase inhibitory activity

**Fig. 3:**
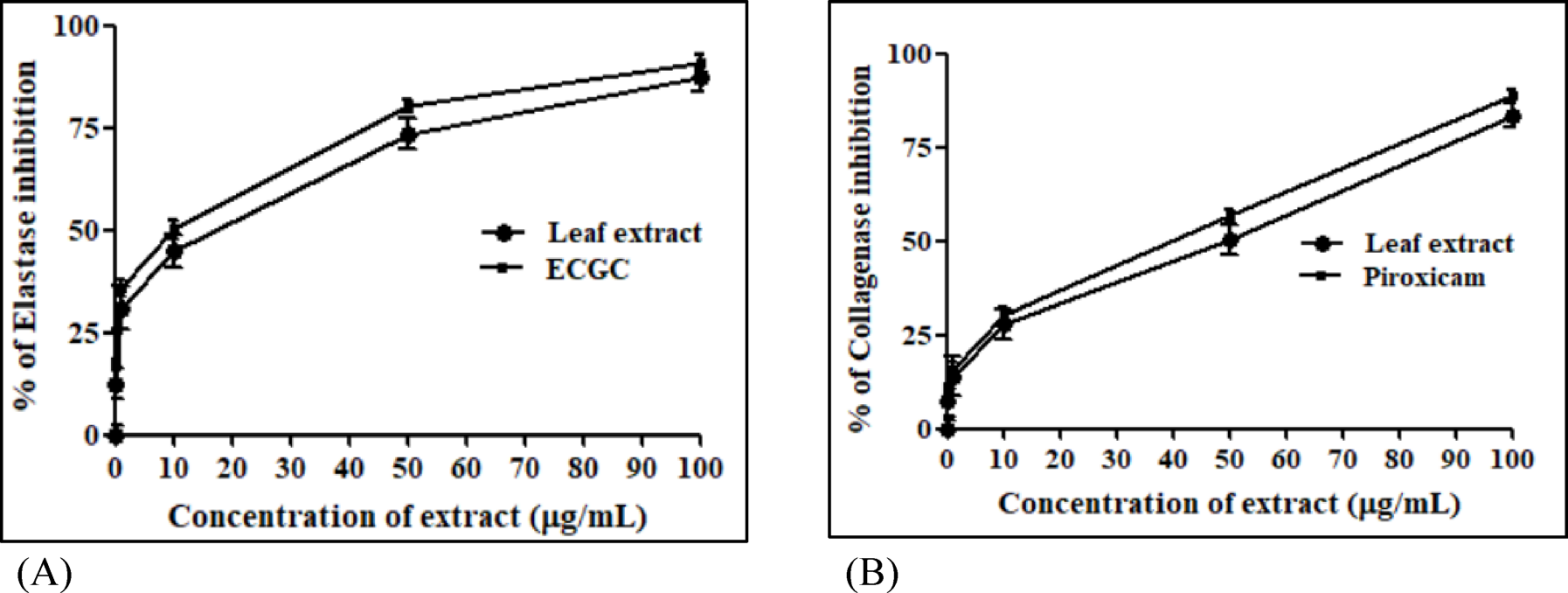
Collagenase (**A**) and elastase (**B**) inhibitory activities of the 80% methanol extract of *S. balansae* leaves.

Biologically, collagenase and elastase enzymes become activated with aging, resulting in reduced skin elasticity, which causes wrinkles or stretchmarks^61^. Thus, the anti-skin aging potential of the extract was evaluated *via* collagenase and elastase inhibitory assays. The results revealed a promising activity of the extract against both enzymes. Different concentrations of the extract were evaluated and showed a dose-dependent inhibition against targeted enzymes as illustrated in Fig. 3A. The leaf extract of *S. balansae* remarkably repressed collagenase activity by IC_50_ value of 48.08 ± 3.42 µg/mL, approaching the value of the positive control, piroxicam (IC_50_= 40.07 ± 2.81 µg/mL). Concerning the elastase assay (Fig. 3B), the extract displayed a promising inhibitory activity with IC_50_ value of 18.02 ± 1.57 µg/mL, compared to standard drug epigallocatechin, EGCG (IC_50_= 10.01 ± 1.07 µg/mL).

Aging process involves synchronization of many complex pathways^62^. Based on the findings of TPC and TFC, the high value of total phenolics and flavonoids might refer to be the main contributor of anti-skin aging due to antioxidant properties which are well-correlated with anti-elastase properties^63^. Since polyphenols possess a complex chemical structure, they display powerful antioxidant activity against ROS, including hydroxyl radicals and superoxide radicals^64^. Moreover, earlier reports proved the ability of natural phenolics to downregulate the proteolytic enzymes *in vitro via* chelation or precipitation^65,66^. Further, the identified components of SBL extract were previously described to employ substantial biological activities beneficial in the cosmetic area such as anti-aging, skin whitening, skin moisturizing, and antioxidant properties. These reported properties energized us to estimate the anti-aging activity of extract using in vitro and in silico molecular modeling experiments.

For instance, some studies proved that quercetin isolated from M. malabathricum leaves was able to exhibit elastase inhibitory activity by 93.86%^67^. Oleanolic acid, a natural triterpenoid of Ligustrum lucidum, was found to inhibit PM10-induced skin aging via suppression of CYP1A1 and tumor necrosis factor–α and interleukin 6 in dermal fibroblasts^68^. Jatrorrhizine, a major alkaloid of Coptis chinensis flowers, was reported to shield against ultraviolet-B-induced oxidative damage^69^. These reports revealed that S. balansae leaves may have potential therapeutic applications against skin wrinkles or aged skin.

### *In silico* molecular docking studies

To ascertain the anti-skin aging potential *of S. balansae* extract, the major components were exposed to *in silico* molecular modeling analysis within the active sites of the target enzymes to evaluate their potential mode of action and binding affinity within the binding sites of the selected enzymes.

#### Assessment of docking affinities with 7EST elastase

**Fig. 4:**
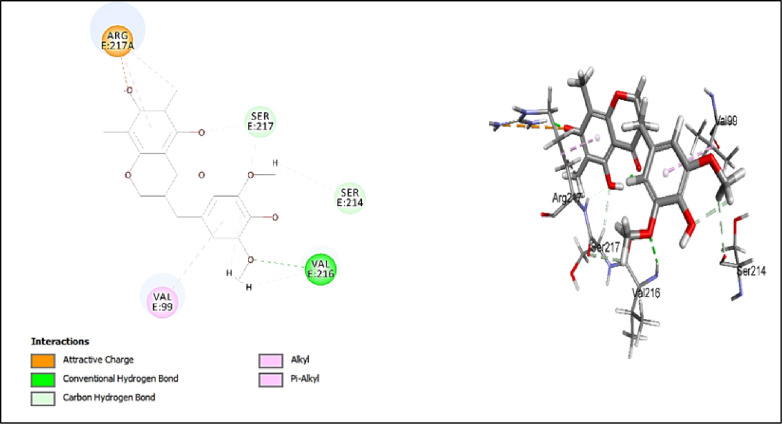
2D and 3D interactions of 5-hydroxy-7,4’-dimethoxy-6, 8-dimethyl homo-isoflavone with 7EST elastase.

**Fig. 5:**
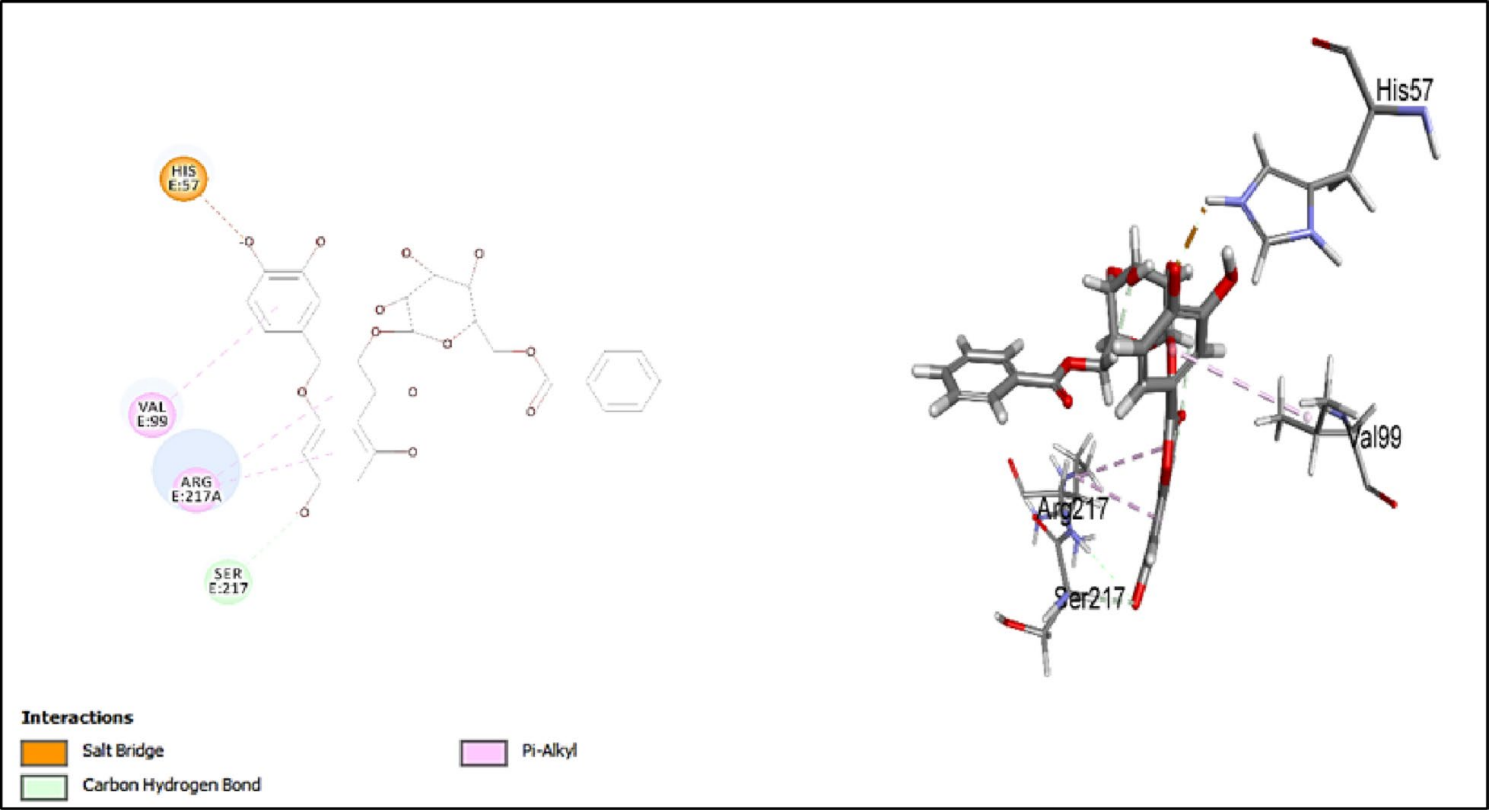
2D and 3D interactions of quercetin-*O*-benzoyl-hexoside with 7EST elastase.

**Fig. 6:**
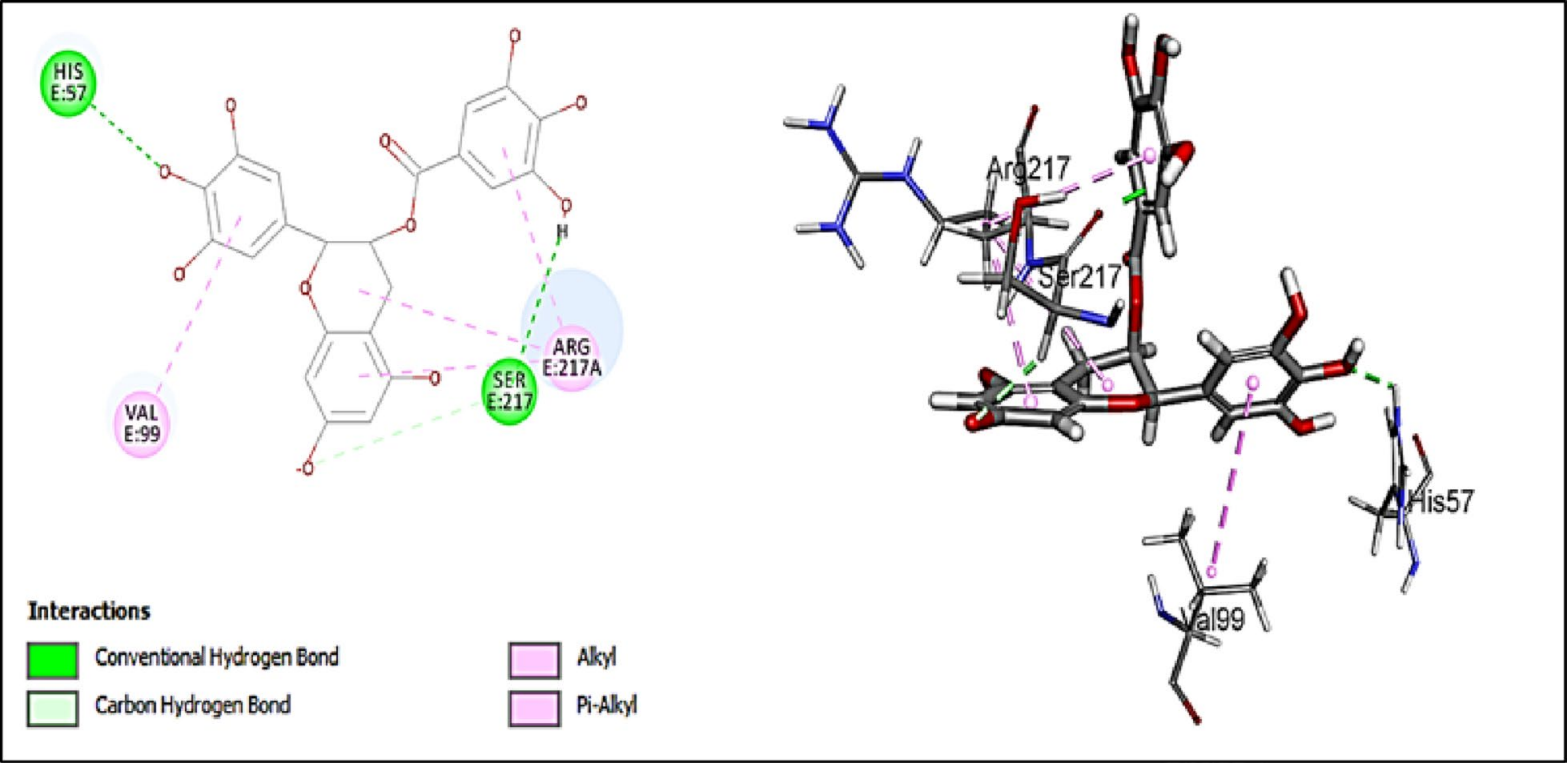
2D and 3D Interactions of EGCG (standard drug) with 7EST elastase.

CDOCKER algorithm was used to dock the tested compounds into the active pocket of 7EST protein (Table 2). The parameters were set to generate ten poses of each compound. The pose with the highest score was selected. This helps to have a glimpse of the binding mode and nature of binding of the investigated compounds. The table below shows the -CDOCKER scores of the investigated compounds. Interestingly, 5-hydroxy-7,4’-dimethoxy-6, 8-dimethyl homo-isoflavone (-CDOCKER score: 38.6753), and quercetin-*O*-benzoyl-hexoside (-CDOCKER score: 37.6048) have the highest -CDOCKER scores. The 2D & 3D interaction diagrams of these compounds are shown in Figs. 4 and 5. The score of 5-hydroxy-7,4’-dimethoxy-6, 8-dimethyl homo-isoflavone might be attributed to conventional hydrogen bond with Val216. In addition, it makes different types of interactions like carbon hydrogen bond, attractive charge, and alkyl interaction but with different amino acids. Quercetin-*O*-benzoyl-hexoside shows salt bridge with HIS57, carbon hydrogen bond with SER217, and *pi*-alkyl interaction with VAL99 & ARG217A. Scopoletin (-CDOCKER score = 22.6619) shows salt bridge with HIS57 and carbon hydrogen interaction with SER195. Justicialoside A shows a lower score of 12.498. Meanwhile, oleanolic acid and eburicoic acid showed low affinities. It was concluded that the amino acid HIS57 is significant in the active site as it is involved in the interaction with all the docked compounds except for 5-hydroxy-7,4’-dimethoxy-6, 8-dimethyl homo-isoflavone. Based on the scores, the first eight compounds contribute to the activity of the extract against elastase enzyme. To further contextualize the in silico findings and draw a direct correlation with the in vitro enzyme inhibition assays, EGCG, the positive control in the elastase assay, was docked into the active pocket of the enzyme (PDB ID:7EST). It showed conventional hydrogen bonds with HIS57 and SER217, alkyl interaction with ARG217A, π-alkyl interactions with VAL99 and ARG217A, and carbon hydrogen bond with SER217 as shown in Fig. 6. In addition, it expressed a high -CDOCKER score of 55.1844 which is notably higher than all investigated metabolites against 7EST. These results are consistent with the experimental data where the standard inhibitor demonstrated greater anti-elastase activity than the extract. Thus, providing validation for the docking protocol in anticipating the relative binding affinities towards this enzyme.

**Table 2:** Docking scores of the major identified compounds with 7EST protein.

Compound name	-CDOCKER score
5-Hydroxy-7,4'-dimethoxy-6, 8-dimethyl homo-isoflavone	38.6753
Quercetin-*O*-benzoyl-hexoside	37.6048
Scopoletin	22.6619
Justicialoside A	12.498
Jatrorrhizine	−5.21591
Oleanolic acid	−52.8138
Eburicoic acid	−86.1852
EGCG (Standard drug)	55.1844

#### Assessment of docking affinities with 2TCL collagenase

**Fig. 7:**
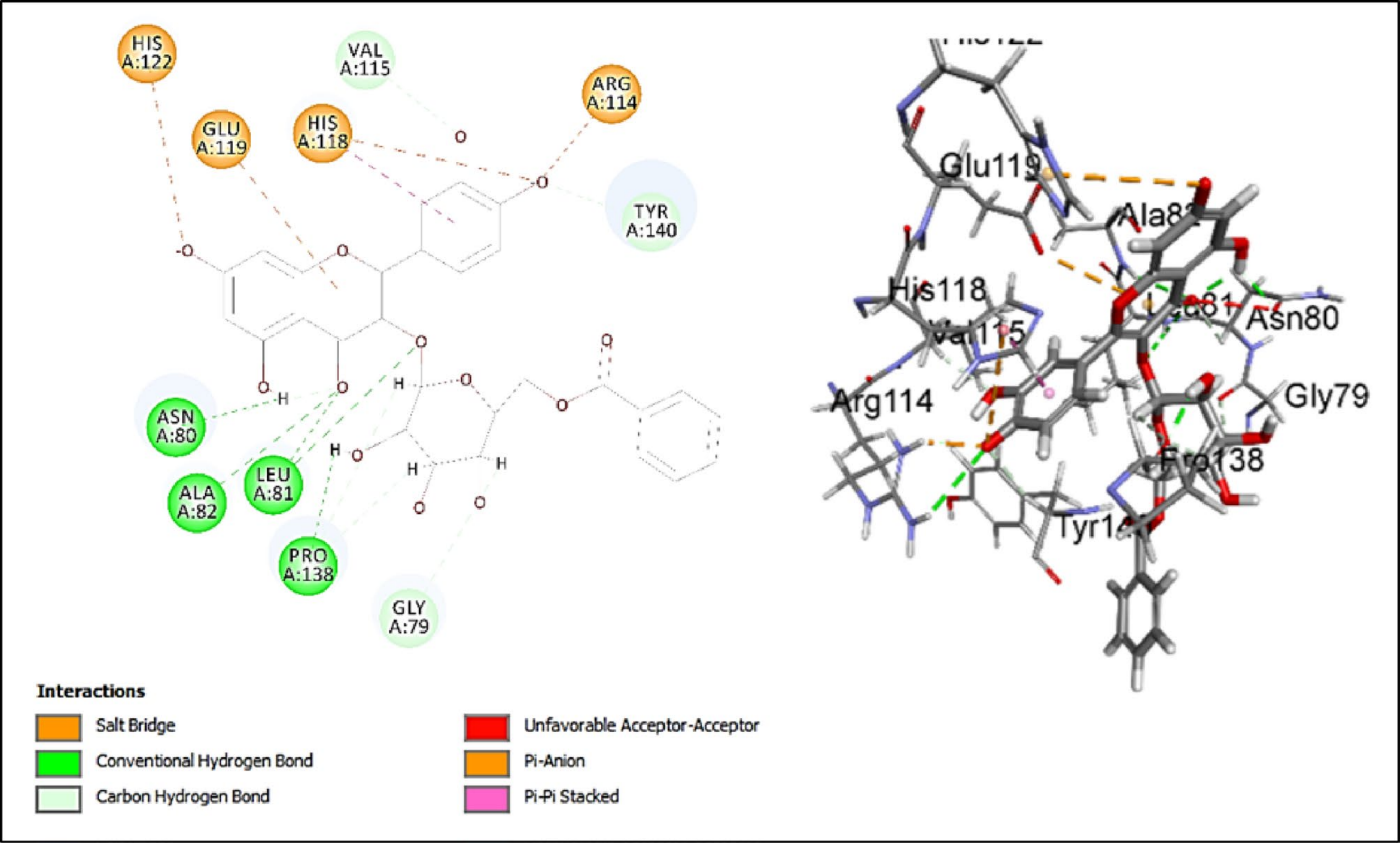
2D and 3D Interactions of quercetin-*O*-benzoyl-hexoside with 2TCL collagenase.

**Fig. 8:**
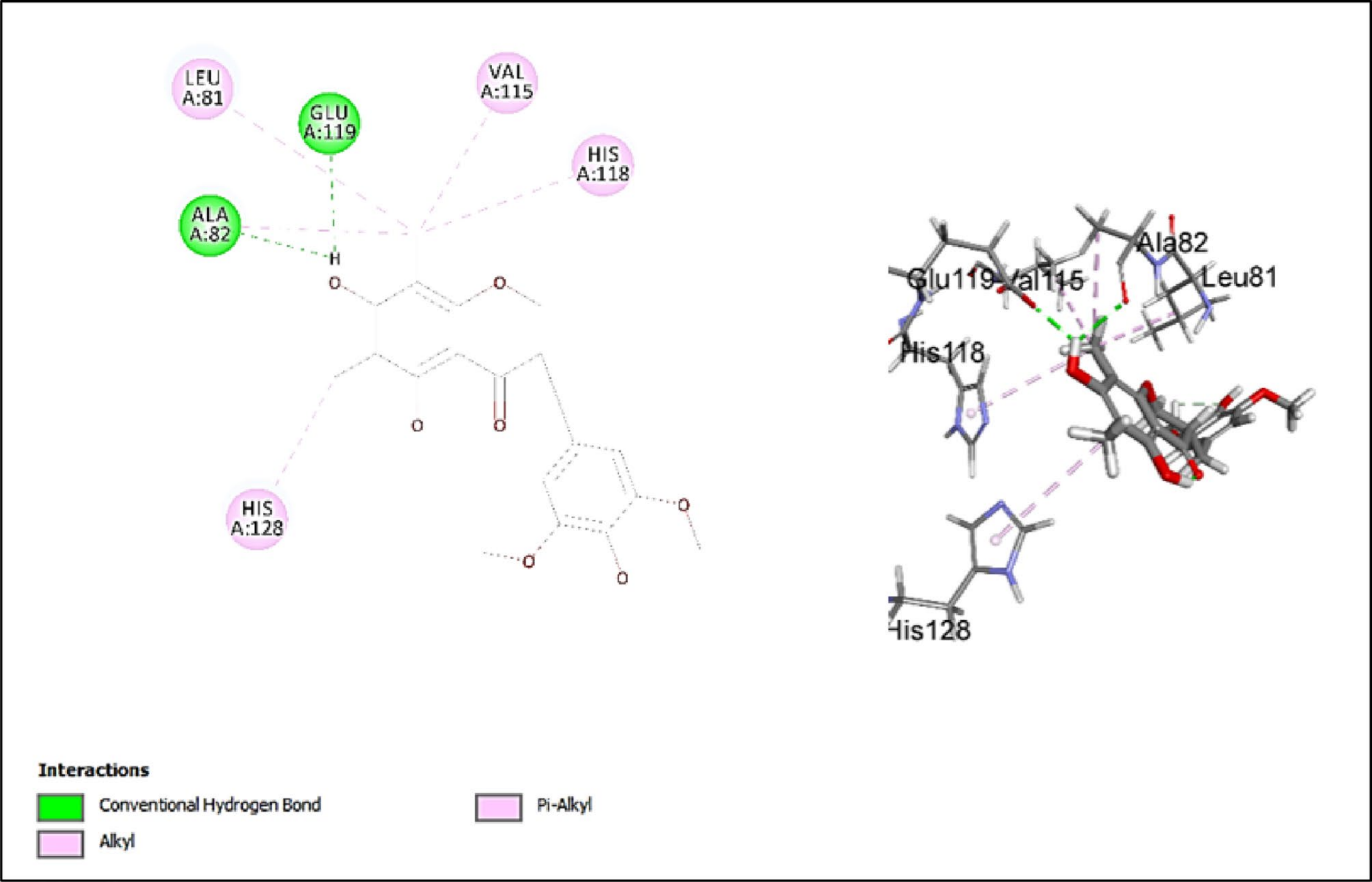
2D and 3D Interactions of 5-hydroxy-7,4’-dimethoxy-6, 8-dimethyl homo-isoflavone with 2TCL collagenase.

**Fig. 9:**
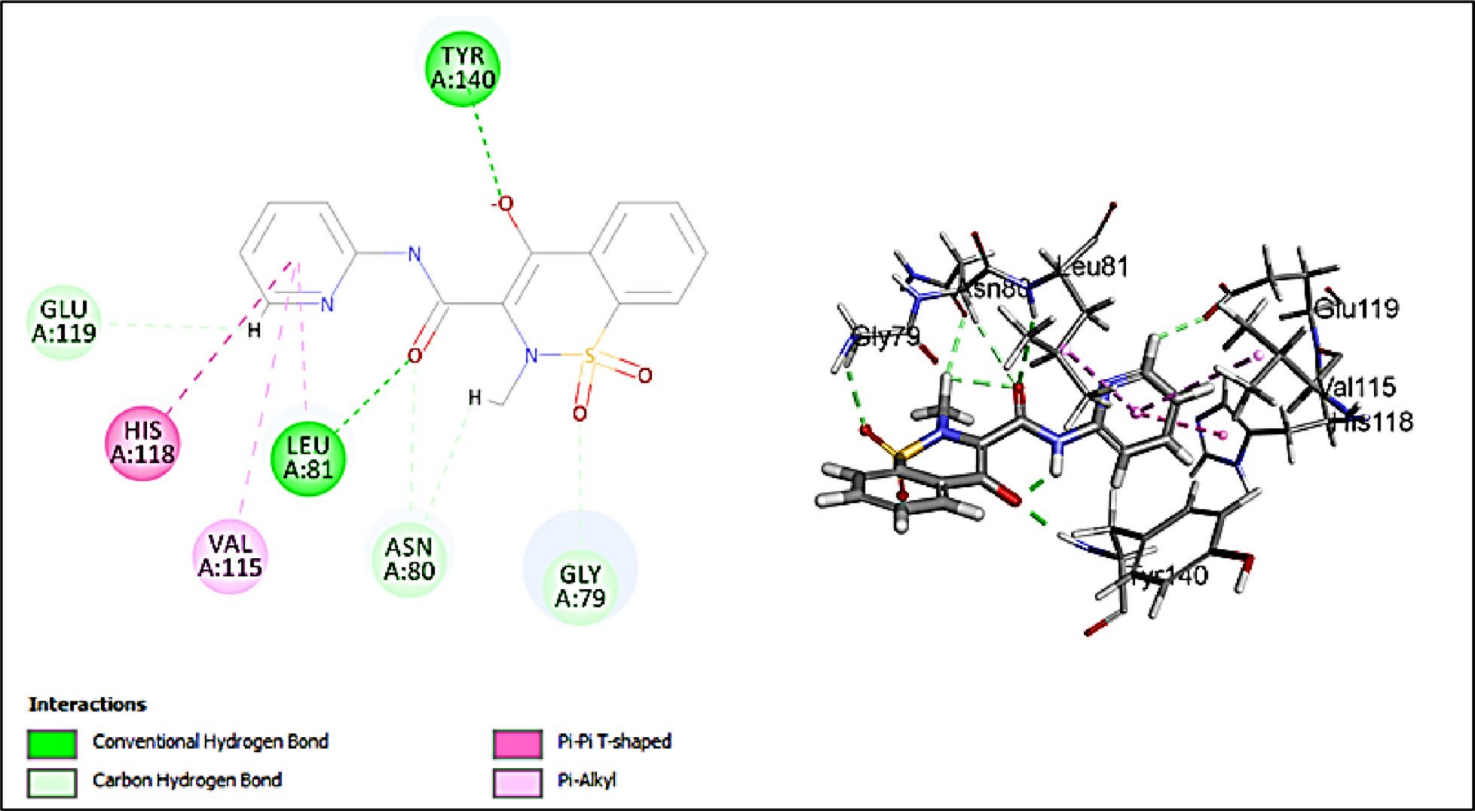
2D and 3D Interactions of piroxicam (standard drug) with 2TCL collagenase.

The major investigated compounds were docked into the active pocket of 2TCL collagenase. Table 3 illustrates the -CDOCKER scores of the docked compounds. Quercetin-*O*-benzoyl-hexoside has the highest score of 28.4866 (Fig. 7). The main interactions are conventional hydrogen bond with ASN80, LEU81, ALA82 and PRO138 and salt bridge with ARG114 and HIS122 and pi-anion interaction with HIS118 and GLU119. 5-Hydroxy-7,4’-dimethoxy-6, 8-dimethyl homo-isoflavone has the highest second score of 26.5854 (Fig. 8). 2D interaction shows conventional hydrogen bond with ALA82 and GLU119. It also shows alkyl and *pi*-alkyl interactions with LEU81, VAL115, HIS118, and HIS128. Scopoletin (-CDOCKER score = 13.1816) shows conventional hydrogen bonds with ALA82 and TYR140 and carbon hydrogen bonds with LEU81 and SER139. Jatrorrhizine has lower score of 10.1764 and the score drops significantly to 2.61696 for justicialoside A. Oleanolic acid and eburicoic acid have very low affinity. The main interactions of the forementioned compound are conventional and carbon hydrogen bonds. All of them make conventional hydrogen bonds except for eburicoic acid. Quercetin-*O*-benzoyl-hexoside is the only compound to make salt bridge and pi-anion which might explain why it has the highest score. LEU81, ALA82, HIS118, and GLU 119 are significant in the active site as they are involved in interactions with many of the tested compounds. It was concluded from the scores that the investigated compounds contribute to the activity of the extract against collagenase enzyme. Further, piroxicam as standard drug, showed conventional hydrogen bonds with LEU81 and TYR140, π-alkyl interaction with VAL115, π- π interaction with HIS118, and carbon hydrogen bond with GLY79, ASN80, andGLU119 (Fig. 9). Although piroxicam was a stronger inhibitor than the extract, its -CDOCKER score (21.3814) was lower than 3 of the annotated compounds. This discrepancy revealed some limitations in the in silico studies where several factors cannot be considered in the docking algorithms such as solubility, bioavailability and enzyme kinetics. Hence, there is a possibility that some docked compounds with higher scores may possess unfavorable physicochemical properties within the experimental conditions or may suffer from poor accessibility to the binding pocket of the enzyme. Additionally, the extract comprises several constituents, therefore, the in vitro activity represents the combined effect of the component compounds which might be synergistic or antagonistic. On the contrary, docking evaluates individual compounds in isolation. Consequently, the highest in vitro inhibitory activity of piroxicam could be accounted for the optimized pharmacodynamic and pharmacokinetic properties as a well-characterized inhibitor.

The in vitro assays demonstrated significant inhibitory activities of the tested extract against the evaluated targets, while the in silico molecular docking studies provided insights into the potential interactions of individual metabolites with the active sites of these enzymes. However, it is important to note that the extract is a complex mixture, and the observed biological effects cannot be attributed solely to the annotated compounds included in the docking analyses. Structural uncertainties of some metabolites, as well as possible synergistic or antagonistic interactions among multiple constituents, limit direct correlation between predicted binding affinities and experimental activities. Therefore, the docking results should be interpreted as supportive mechanistic insights rather than definitive explanations of the observed in vitro effect.

**Table 3:** Docking scores of the major identified compounds with 2TCL protein.

Compound	-CDOCKER score
Quercetin-*O*-benzoyl-hexoside	28.4866
5-Hydroxy-7,4’-dimethoxy-6, 8-dimethyl homo-isoflavone	26.5854
Scopoletin	13.1816
Jatrorrhizine	10.1764
Justicialoside A	2.61696
Oleanolic acid	−60.232
Eburicoic acid	-−92.6255
Piroxicam (standard drug)	21.3814

## Conclusion

The phytochemical analysis of *S. balansae* leaf extract revealed a diverse array of bioactive compounds, including quercetin-*O*-benzoyl-hexoside, 5-hydroxy-7,4’-dimethoxy-6,8-dimethyl homo-isoflavone, and scopoletin. The extract exhibited high total phenolic and flavonoid contents, demonstrating significant antioxidant activity and potent inhibition of collagenase (IC_50_= 48.08 µg/mL) and elastase (IC_50_= 18.02 µg/mL). In silico docking studies provided further evidence of the strong molecular interactions of these compounds with enzymes linked to skin aging, underscoring their therapeutic potential. These findings highlight the promise of *S. balansae* leaf extract as a natural source of bioactive compounds with potential applications in anti-aging formulations. Further research needs to be conducted on safety and toxicological studies, clinical validation and mechanistic studies to support its use in cosmeceutical innovations.

## Electronic Supplementary Material

Below is the link to the electronic supplementary material.

Supplementary Material 1.

## Data Availability

All data supporting this study are provided in the manuscript and/or supplementary file.
