# Supplementary material for: UPLC-ESI/MS-MS-based phytochemical analysis of Schinopsis balansae leaf extract and anti-skin ageing properties supported with in silico molecular docking experiments
Source: Sci Rep. 2026 May 15;16:15219. doi: 10.1038/s41598-026-50709-6 (PMC13179384; doi:10.1038/s41598-026-50709-6)
Supplement: Supplementary file 1 — Supplementary Material 1. [file 41598_2026_50709_MOESM1_ESM.docx]

**SUPPLEMENTARY MATERIAL**

**UPLC-ESI/MS-MS based Phytochemical Analysis of *Schinopsis balansae* Leaf Extract and Anti-skin Ageing Properties Supported with *In silico* Molecular Docking Experiments**

Heba A. S. El-Nashar^1,2^*, Ayman M. Al-Qaaneh^3^, Mahmood A. Al-Azzawi^4^*, Abdalrahman Tarek^5^, Esraa A. Elhawary ^1^, Naglaa S. Ashmawy^1,6^*

*^1^Department of Pharmacognosy, Faculty of Pharmacy, Ain Shams University, Abbassia 11566 Cairo, Egypt*

*^2^Department of Pharmacognosy, Faculty of Pharmacy, Modern University for Technology & Information, Cairo, Egypt.*

*^3^ Faculty of Allied Medical Sciences, Al-Balqa Applied University (BAU), Al-Salt 19117, Jordan*

*^4^Department of Forensic Science, College of Science, Al-Karkh University of Science, Baghdad P.O. Box 10081, Iraq*

*^5^Department of Pharmaceutical Chemistry, Faculty of Pharmacy, Ain Shams University, Abbassia 11566 Cairo, Egypt*

*^6^ Department of Pharmaceutical Sciences, College of Pharmacy, Gulf Medical University, P.O. Box 4184, Ajman, United Arab Emirates*

***Corresponding authors:** Heba A.S. El-Nashar: [heba_pharma@pharma.asu.edu.eg](mailto:heba_pharma@pharma.asu.edu.eg) , Mahmood A. Al-Azzawi: [mmahmood41@yahoo.com](mailto:mmahmood41@yahoo.com), Naglaa S. Ashmawy: [naglaa.saad@pharma.asu.edu.eg](mailto:naglaa.saad@pharma.asu.edu.eg)


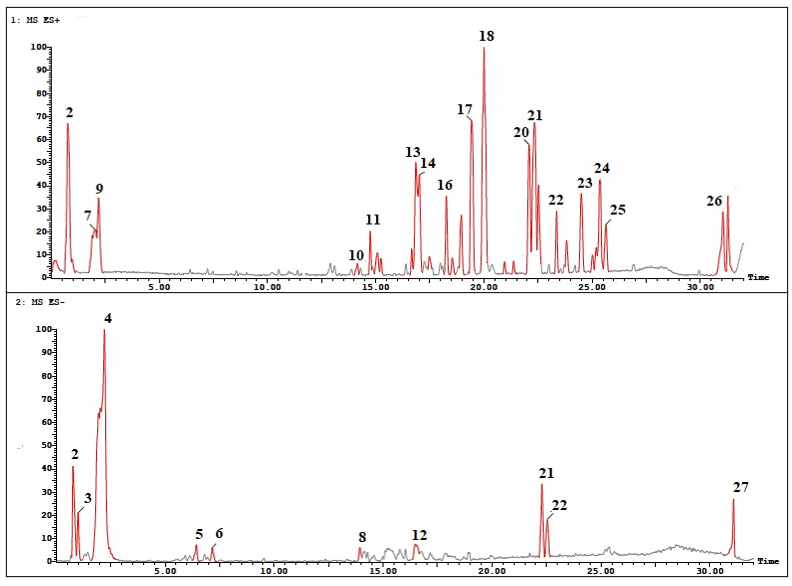


(**A**)

(**B**)

**Figure S1.** Total ion chromatogram (TIC) of methanol extract of *S. balansae* leaves in positive ion mode (**A**) and negative ion mode (**B**).

**
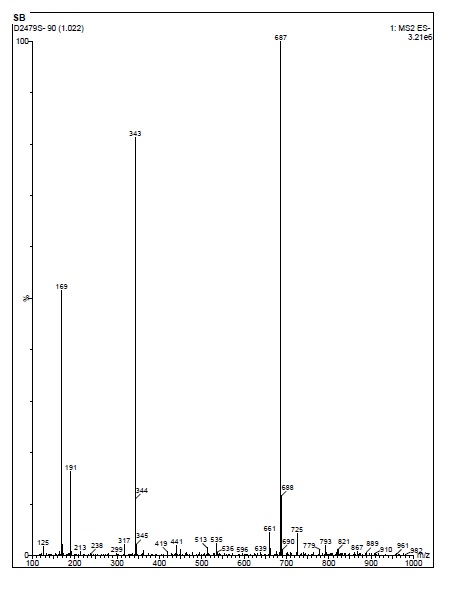
**

**Figure S2**. MS/MS fragmentation pattern of 5,6-dihydroxy-3',4',7-trimethoxy flavone

**
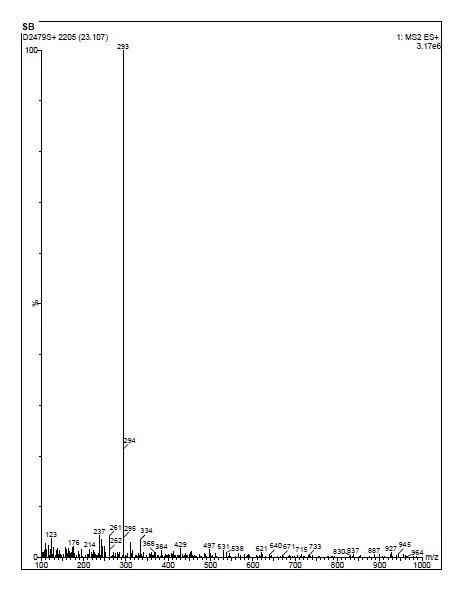
**

**Figure S3**. MS/MS fragmentation pattern of justicialoside A

**
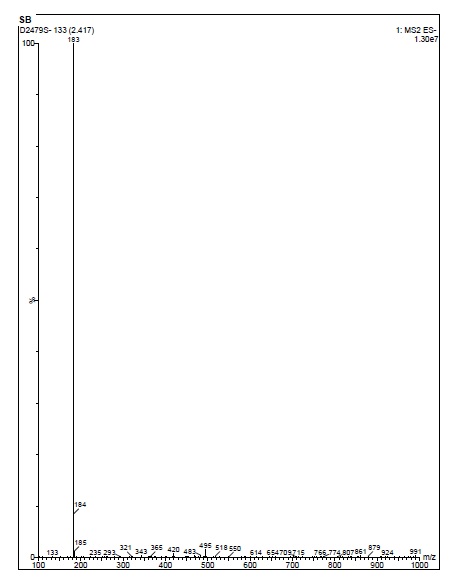
**

**Figure S4**. MS/MS fragmentation pattern of malonyl-coumaroyl-quinic acid

**
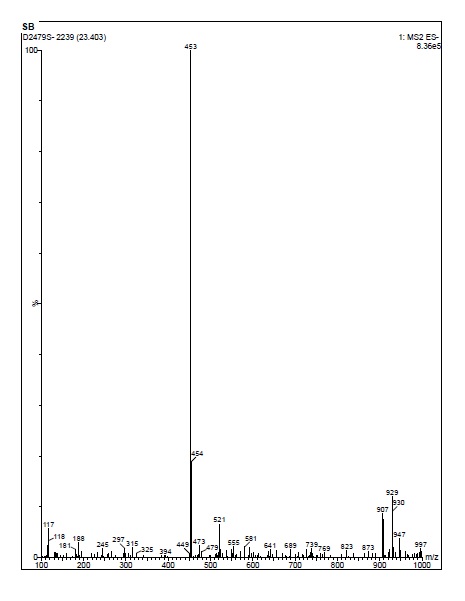
**

**Figure S5**. MS/MS fragmentation pattern of oleanolic acid

**
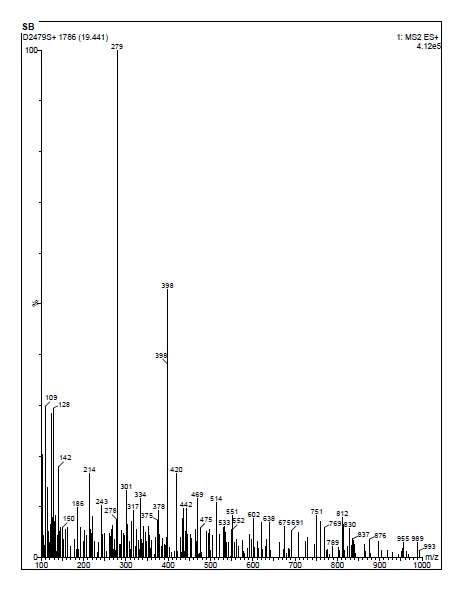
**

**Figure S6**. MS/MS fragmentation pattern of eburicoic acid
